# The Essential Rot1 Protein Links Glycosylation, Cell Wall Integrity, and Pathogenic Development in *Candida albicans*

**DOI:** 10.3390/jof12040244

**Published:** 2026-03-26

**Authors:** Anna Janik, Ewa Zatorska, Urszula Perlińska-Lenart, Sebastian Piłsyk, Joanna S. Kruszewska

**Affiliations:** Institute of Biochemistry and Biophysics Polish Academy of Sciences, Pawinskiego 5a, 02-106 Warsaw, Poland; annaj@ibb.waw.pl (A.J.); zatorska.ewa@gmail.com (E.Z.); lenart@ibb.waw.pl (U.P.-L.); seba@ibb.waw.pl (S.P.)

**Keywords:** *Candida albicans*, Rot1 protein, glycosylation, filamentation, cell wall formation

## Abstract

The Rot1 protein is a chaperone involved in glycosylation, dolichol phosphorylation, cell wall synthesis, and protein folding in the yeast *Saccharomyces cerevisiae*. Available information on cell wall defects in the *S. cerevisiae rot1-1* mutant and the association of Rot1 with protein glycosylation suggest that in the case of *Candida albicans*, Rot1 may be involved in pathogenesis, since both cell wall synthesis and protein glycosylation are closely related to the formation of pathogenic structures in *C. albicans*. As Rot1 has not been found in humans, it seems particularly attractive for study in the context of *C. albicans* pathogenicity. This protein takes on additional significance because deletion of the gene that encodes Rot1 is lethal for yeast. In this study, we cloned and analyzed the function of the candidate protein CaRot1 from *C. albicans* in the *S. cerevisiae rot1Δ*/*ROT1* mutant. Furthermore, we investigated the consequences of restricted Ca*ROT1* expression in *C. albicans*. We have shown that a low amount of Rot1 limits the transfer of oligosaccharide to protein, inhibits the activity of the first steps of oligosaccharide formation on dolichyl diphosphate, changes the composition of the cell wall, limits the protection of *C. albicans* against ER and abiotic stress, and finally prevents filamentation, which is an invasive structure of *C. albicans*.

## 1. Introduction

The Target of Rapamycin (TOR) signaling pathway is a central regulator of eukaryotic cell growth and metabolism in response to nutrient availability. In *Saccharomyces cerevisiae*, *TOR2* is essential for cell viability and plays a key role in coordinating cytoskeletal organization, cell wall integrity, and polarized growth, particularly under nitrogen-dependent conditions [1]. *ROT1* (Reverse Of TOR) was originally identified as a multicopy suppressor of *TOR2* lethality, suggesting a functional connection between Rot1 and pathways regulating cell growth and homeostasis.

Rot1 is an essential endoplasmic reticulum (ER)-localized protein in *S. cerevisiae* implicated in several fundamental cellular processes, including β-1,6-glucan synthesis, actin cytoskeleton dynamics, and the lysis of autophagic bodies [2,3,4]. In addition, Rot1 has been proposed to function as an ER chaperone involved in protein folding [5]. Our previous studies demonstrated that *ROT1* acts as a multicopy suppressor of the temperature-sensitive phenotype of the *sec59-1* mutant [6], indicating a link between Rot1 and protein glycosylation. The *SEC59* gene encodes dolichyl kinase, which catalyzes the phosphorylation of dolichol to dolichyl phosphate—an indispensable lipid carrier for oligosaccharide assembly during *N*-glycosylation.

Further evidence supporting a direct role of Rot1 in protein glycosylation comes from its physical interaction with Ost3, a subunit of the oligosaccharyltransferase (OST) complex responsible for transferring oligosaccharides from dolichyl diphosphate to nascent polypeptides during *N*-linked glycosylation [7]. The OST complex consists of eight subunits and exists in two isoforms containing either Ost3 or its homolog Ost6 [8,9,10]. While both Ost3 and Ost6 are nonessential oxidoreductases, the Ost3-containing complex is required for efficient glycosylation of a broader subset of proteins [11]. Importantly, Rot1 interacts specifically with Ost3 but not with Ost6, indicating functional specialization within OST isoforms [7]. Since *N*- and *O*-glycosylation are functionally linked and occur during translocation of nascent polypeptides into the ER lumen [12], Rot1 has also been implicated in *O*-mannosylation, although the underlying mechanism remains unclear [7].

In addition to its role in glycosylation, Rot1 is required for proper cell wall assembly. Limiting *ROT1* expression in *S. cerevisiae* leads to cell aggregation associated with decreased β-1,6-glucan levels and compensatory increases in chitin and β-1,3-glucan content [13]. These findings suggest that Rot1 influences cell wall architecture and may indirectly affect pathways targeted by antifungal drugs, such as β-1,3-glucan synthesis inhibited by echinocandins. Notably, in the pathogenic yeast *Candida albicans*, alterations in glycosylation and dolichol metabolism profoundly affect virulence-associated traits, including filamentation and biofilm formation [14,15,16,17].

Despite its essential role in *S. cerevisiae*, the function of Rot1 in pathogenic fungi has not been characterized. Importantly, Rot1 has no homolog in humans, making it an attractive candidate for antifungal research. In this study, we cloned and functionally characterized the *C. albicans* homolog of *ROT1* (Ca*ROT1*) using heterologous complementation in an *S. cerevisiae rot1Δ*/*ROT1* mutant. We further investigated the effects of restricted Ca*ROT1* expression in *C. albicans*, focusing on protein glycosylation, cell wall composition, filamentation, and sensitivity to ER and abiotic stresses. Our results reveal Rot1 as a central regulator linking glycosylation metabolism, cell wall integrity, and pathogenic development in *C. albicans*.

## 2. Materials and Methods

### 2.1. Strains and Growth Conditions

*S. cerevisiae* strain BY4743; (genotype: MATa/MATα; his3Δ1/his3Δ1; leu2Δ0/leu2Δ0; LYS2/lys2Δ0; met15Δ0/MET15; ura3Δ0/ura3Δ0; YMR200w/YMR200w::kanMX4).

*C. albicans* strain CAI4 (genotype: *ura3 ∆::imm434*/*ura3 ∆::imm434*), a uridine auxotroph, was used for deletion of the *ROT1* gene.

*E. coli* strain DH5α F’ (genotype: F’ supE44 ∆lacU169 {Φ80 lacZ ∆M15} hsdR17 recA1 endA1 ngyrA96 thi-1 relA1) was used for plasmid propagation.

*S. cerevisiae* strains were grown at 30 °C in SD medium (0.67% yeast nitrogen base, 2% glucose) with the necessary supplements. For sporulation, 1%CH_3_COOK pH 7 and 2% agar were used. Galactose was used at 2% as a carbon source.

*C. albicans* strains were routinely grown at 30 °C in YPD medium (1% yeast extract, 1% Bacto-peptone, 2% glucose) or SD medium. Uridine auxotrophic strains were grown on media supplemented with uridine (40 µg/mL).

The ability to form hyphae was tested on Spider medium (1% nutrient broth, 1% mannitol, 0.2% K_2_HPO_4_, and 1.35% agar) and YP-Serum (1% yeast extract, 0.5% peptone, 10% horse serum, and 2% agar) [16].

### 2.2. Phenotype Analysis

To test yeast strains for sensitivity to various compounds, 3 µL of serial 1:10 dilutions (starting at 1 × 10^7^ cells) of exponentially growing cultures were spotted on YPD agar plates supplemented with uridine and indicated doses of various agents and incubated for 72 h at 30 °C.

Strains were cultivated on 96-well plates using a Varioskan LUX Multimode Microplate Reader (Thermo Fisher Scientific, Waltham, MA, USA). Cell density was measured every hour in OD_600_.

For sensitivity assays, solid YPD medium (1.5% agar) was supplemented with doxycycline (40 µg/mL), tunicamycin (1 µg/mL), or Congo Red (5, 10, 15 µg/mL), Calcofluor White (5, 10, 15 µg/mL), or DTT (30 mM). The nitrogen starvation medium contained 0.17% yeast nitrogen base without ammonium sulfate and amino acids, 2% glucose, and 5 μM ammonium sulfate [18].

### 2.3. CaROT1 Expression in S. cerevisiae

The Ca*ROT*1 gene was amplified using the ROT1orfF and ROT1orfR primers (Table S1) and cloned into the pESC-ura vector (Stratagene) under the control of the GAL1 promoter at cloning site II following digestion with BamHI and NheI. The diploid *Saccharomyces cerevisiae* strain *rot1Δ*/*ROT1*, carrying a deletion of one copy of the Sc*ROT1* gene, was transformed with the *Candida albicans CaROT1* gene using the one-step transformation method described by Chen et al. [19], and transformants were selected on SD medium. Subsequently, transformants were cultivated on sporulation medium, and tetrads were dissected. Site-directed mutagenesis of the *CTG* codon to *TCG* was performed using mutROT1F and mutROT1R primers and the QuickChange II Site-Directed Mutagenesis Kit (Agilent Technologies, Santa Clara, CA, USA) according to the manufacturer’s standard protocol.

### 2.4. Construction of C. albicans ROT1 Deleted Strain

One copy of *ROT1* (*orf1*9.6029) was deleted using the “URA-Blaster” method [20]. For the construction of the deletion cassette, the following primers were used: ROT1-flank1-Fr1F/ROT1-flank1-Fr1R for amplification of the 5′ region of homology (−311 upstream of the ORF to +61 downstream of the AUG start codon) and ROT1-flank2-Fr2F/ROT1flank2-Fr2R for amplification of the 3′ region of homology (+623 to +937 downstream of the AUG start codon) (Table S1).

The obtained fragments were cloned to the p5921 plasmid in the SacI/BglII and SalI/PstI sites, respectively. The SacI/PstI fragment was then excised and used for gene replacement in the CAI4 strain to obtain the rot1*∆::hisG-URA3-hisG*/*ROT1* strain. To induce the excision of the *URA3* gene, *C. albicans* transformants were grown on FOA plates (SD medium with 0.3% 5-fluoroorotic acid) [20].

To put the second copy of *ROT1* under the control of the tetracycline promoter (TRp), primers ROTtetF and ROTtetR were used to amplify the cassette on the template of the p2151c plasmid. The 4092 bp fragment contained: a 67 bp fragment homologous to region −359 to −292 bp upstream of the *ROT1* start codon, the *URA3* selection marker, the fusion transactivator–*tetR-ScHAP4AD*, the regulatable *tetO-ScHOP1* promoter, and a 66 bp sequence homologous to region −1 to +65 bp of *ROT1*. The cassette was used for transformation of the *rot1∆::hisG*/*ROT1* strain.

Proper construction of the strains was confirmed by Southern blot analysis (Figure S1).

### 2.5. Quantitative Reverse Transcription PCR (RT-qPCR)

Total RNA was obtained from *rot1∆*/*ROT1* and *rot1∆*/*TR*p*ROT1* mutants and the CAI4 parental strain using the single-step method described by Chomczynski and Sacchi [21]. Reverse transcription was performed using the iScript Advanced cDNA Synthesis Kit for RT-qPCR (Bio-Rad Laboratories, Inc, Hercules, CA, USA) and standard protocol. qPCR assays were performed in a Light Cycler 1.6 Instrument (Roche Life Science, Basel, Switzerland). For the amplification, Light Cycler Fast Start DNA Master PLUS SYBR Green (Roche Life Science) mix was combined with 0.2 μM forward and reverse primers (Table S1) and cDNA diluted 1:5 with nuclease-free water. The thermal cycling conditions were as follows: initial denaturation at 95 °C for 10 min, followed by 40 cycles of denaturation at 95 °C for 10 s, annealing at 58 °C for 10 s, and elongation at 72 °C for 1 s per 25 bp.

The relative expression software tool REST-MCS ©-version 2 [22] was used to quantitate the relative mRNA levels of the selected genes. Data normalization was carried out against the transcript of a reference actin gene.

### 2.6. Cell Membrane Preparation

*C. albicans* strains were cultured to OD_600_ = 1–1.5; the cells were then harvested by centrifugation and resuspended in 2 volumes of 50 mM Tris/HCl buffer pH 7.4 containing 15 mM MgCl_2_ and 9 mM 2-mercaptoethanol. The suspension was homogenized with 0.5 mm glass beads, and the homogenate was centrifuged at 4000× *g* for 10 min to remove unbroken cells and cell debris. The supernatant was centrifuged for 1 h at 50,000× *g*, and the pelleted membrane fraction was used for enzymatic assay.

### 2.7. N-Acetylglucosamine Transferase Activity Assay

The activity was measured in the membrane fraction by incubation (final volume 50 µL) of 250 µg of membrane protein with 1 × 105 cpm of UDP-*N*-acetyl-D-glucosamine [glucosamine-^14^C(U)] (sp. act. 300 Ci/mol, American Radiolabeled Chemicals, Inc., St. Louis, MO, USA) in 40 mM Tris-HCl, pH 7.4, 10 mM MgCl_2_, and 0.1% Nonidet NP-40 at 30 °C for 30 min [23]. The reaction was stopped by the addition of 4 mL of chloroform:methanol (3:2, *v*/*v*), and the mixture was washed once with 1/5 volume of 4 mM MgCl_2_ and twice with 4 mM MgCl_2_ in chloroform:methanol:water (3:48:47, *v*/*v*/*v*). Radioactive dolichyl diphosphate *N*-acetylglucosamine and dolichyl diphosphate chitobiose were measured in a scintillation counter.

### 2.8. Determination of N-Acetylglucosaminidase (HexNAcase) Activity In Situ

The HexNAcase in situ activity staining was performed as described [24]. To induce HexNAcase production, strains were grown for 16 h in SC medium supplemented with 25 mM *N*-acetylglucosamine (GlcNAc) in the presence or absence of doxycycline. Cells were disrupted in 10 mM Tris/HCl, pH 8, containing protease inhibitor cocktail (Sigma-Aldrich, Ipswich, MA, USA). For endoglycosidase H (Endo H) treatment, the native sample was treated with 25 milliunits of EndoH (New England Biolabs, Ipswich, MA, USA) for 16 h at 37 °C in 50 mM sodium acetate, pH5.2. Samples were mixed with native loading dye and run on a Tris/acetate 3–8% gradient polyacrylamide gel (Invitrogen, Thermo Fisher Scientific, Waltham, MA, USA) under non-denaturing conditions. The gel was washed in 0.1 M citrate/KOH buffer, pH 4, for 10 min at room temperature and then incubated in substrate solution (0.18 mM naphthyl-GlcNAc (Glyco-synth Ltd., Warrington, UK) in 0.1 M citrate/KOH buffer, pH 4) for 30 min at 37 °C. The reaction was visualized by incubation in the substrate solution plus 0.7 mM Fast Blue at 60 °C until the color developed.

### 2.9. Cell Wall Preparation

*C. albicans* was cultivated in YPD medium, washed with 10 mM Tris/HCl, pH 7.5, suspended in the same buffer, disintegrated with 0.5 mm glass beads, and centrifuged at 1500× *g* for 10 min. The resulting pellet containing cell walls was washed with ice-cold 1 M NaCl until the disappearance of absorbance at 260–280 nm [25].

### 2.10. Determination of Cell Wall Carbohydrates

Lyophilized cell wall was hydrolyzed o/n in 4 M trifluoroacetic acid (TFA) at 100 °C. After cooling on ice, samples were centrifuged at 17,000× *g* for 5 min at 4 °C. The supernatant was dried under nitrogen and washed twice with pure methanol. After removing methanol with nitrogen, the pellet was resuspended in miliQ water and purified on a Millipore Filter Device (0.45 µm pores) by centrifugation at 16,000× *g* for 4 min. Monosaccharides were determined by high-performance anion-exchange chromatography using a Dionex ICS-3000 Ion Chromatography System with a Carbo Pac PA10 analytical column (Thermo Fisher Scientific, Waltham, MA, USA). Neutral sugars were eluted with 18 mM NaOH at 0.25 mL/min [26].

### 2.11. Determination of Polysaccharides

The amount of glucans in the cell was determined as described previously [25], with a slight modification. For quantification of alkali-soluble-1,6 β-glucan, cell walls (200 mg) were suspended in 3% NaOH, heated at 75 °C for 1 h, and centrifuged. The supernatant was dialyzed overnight at 4 °C against distilled water and lyophilized, and the amount of alkali-soluble-1,6-β-glucan was estimated by the method described by Dubois et al. [27]. The remaining pellet was washed twice with 0.1 M Tris-HCl, pH 7.4, and once with 10 mM Tris-HCl, pH 7.4, and digested overnight with zymolyase 20T (5 mg/mL in 10 mM Tris-HCl, pH 7.4; ICN Biomedicals Inc., Costa Mesa, CA, USA). Then the samples were centrifuged (13,000 rpm; 15 min), and the supernatant was used to estimate the amount of alkali-insoluble-1,3-β-glucan by the same method. The remaining pellets were incubated for 16 h with 70% sulfuric acid at 4 °C and then diluted 10-fold with water and heated to 100 °C for 8 h. After being neutralized with 2 M NaOH, the samples were used to estimate the amount of alkali-insoluble-1,3-β-glucan.

The level of chitin was measured according to Bulik et al. [28].

## 3. Results

### 3.1. Functional Identification of orf19.6029 as CaROT1 Using S. cerevisiae rot1Δ/ROT1 Mutant

To determine whether *C. albicans* possesses a functional homolog of Rot1, a homology search of the *C. albicans* genome database (Candida Genome Database; http://www.candidagenome.org/, accessed on 8 June 2025) was performed. This analysis identified orf19.6029 as a candidate *ROT1* homolog.

**Figure 1:**
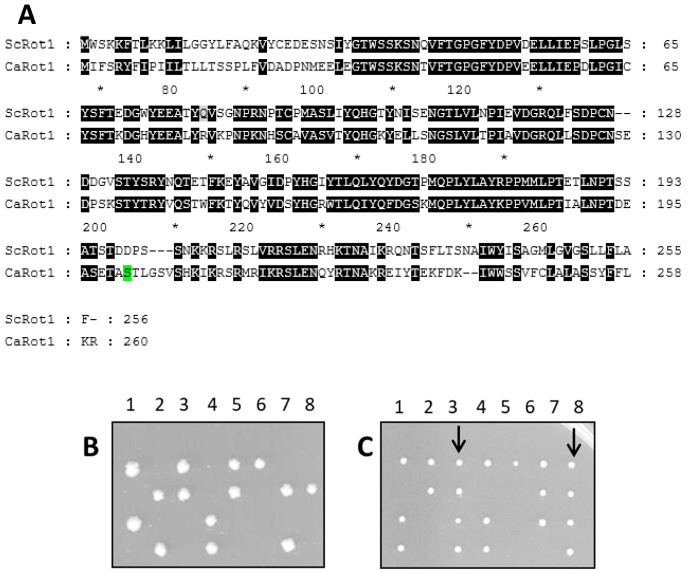
Sequence comparison of Rot1 proteins and functional complementation analysis. (**A**) Amino acid sequence alignment of Rot1 from *Saccharomyces cerevisiae* (ScRot1) and its homolog from *Candida albicans* (CaRot1). (**B**,**C**) Tetrad dissection analysis of *S. cerevisiae rot1Δ*/*ROT1* diploid strains transformed with plasmids expressing Ca*ROT1* (**B**) or Ca*ROT1^TCG^* (**C**). Arrows indicate viable spores recovered after sporulation. The * are amino acid numbers. Instead of every 10, numbers are shown every 20, and every 10 is replaced with an asterisk.

Sequence analysis of the 783 bp open reading frame (ORF) of orf19.6029 revealed that it encodes a protein of 260 amino acids. The predicted protein shares 57% amino acid identity with *S. cerevisiae* Rot1 (ScRot1), indicating a high degree of evolutionary conservation (Figure 1A).

Notably, the sequence contains a single *CTG* codon, which in *C. albicans* is translated as serine, whereas in *S. cerevisiae* the *CTG* codon specifies leucine (Figure 1A in green). This codon reassignment is known to complicate heterologous expression of *Candida* genes in *S. cerevisiae*.

To assess the functionality of orf19.6029, the native gene was introduced into an *S. cerevisiae rot1Δ*/*ROT1* diploid strain. Following sporulation and tetrad dissection, only two viable spores were recovered (Figure 1B), indicating that the native *Candida* gene was unable to complement the lethal *rot1Δ* mutation in *S. cerevisiae*. This result suggested that orf19.6029, as expressed in *S. cerevisiae*, did not provide functional Rot1 activity.

Given the presence of the *CTG* codon, we hypothesized that mistranslation of serine to leucine during heterologous expression could alter protein structure and compromise function. To test this possibility, the *CTG* codon in orf19.6029 was replaced with *TCG*, a standard serine codon in *S. cerevisiae*. The resulting allele, designated Ca*ROT1^TCG^*, was transformed into the *S. cerevisiae rot1Δ*/*ROT1* strain.

Following sporulation and tetrad dissection, four viable spores were recovered from diploids expressing Ca*ROT1^TCG^* (Figure 1C, lines 3 and 8), demonstrating successful complementation of the lethal *rot1Δ* mutation.

These results confirm that orf19.6029 encodes a functional homolog of Rot1 and that correct decoding of the *CTG* codon is essential for CaRot1 activity in *S. cerevisiae*.

To investigate the function of Rot1 in *C. albicans*, we generated a conditional mutant in which one copy of orf19.6029 (Ca*ROT1*) was deleted, and the second copy was placed under the control of a tetracycline-regulatable promoter (*TR*p*ROT1)* to generate the *rot1Δ*/*TR*p*ROT1* strain. Genomic modifications in the *C. albicans* CAI4 background were confirmed by Southern blot analysis (Figure S1).

### 3.2. Disruption of the CaROT1 Gene in C. albicans

To quantify Ca*ROT1* expression in the wild type and the mutant strain, total RNA was isolated and reverse transcription qPCR analysis was performed.

**Figure 2:**
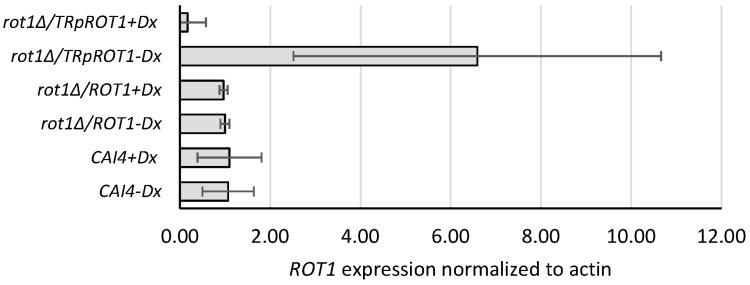
Transcript level of Ca*ROT1* in *C. albicans* determined by RT-qPCR. *rot1∆*/*ROT1*-hemizygous strain; *rot1Δ*/*TR*p*ROT1*-conditional mutant carrying *ROT1* under tetracycline-repressible promoter; CAI4-parental control strain. Cultures were grown for 16 h in YPD, and strains were cultivated with (+Dx) or without (−Dx) doxycycline. Data represent mean ± SD from three independent experiments, each performed in triplicate.

Significantly reduced Ca*ROT1* expression was observed in the *rot1Δ*/*TR*p*ROT1* strain grown in the presence of doxycycline (+Dx), whereas cultivation in the absence of doxycycline (−Dx) resulted in gene overexpression (Figure 2). In contrast, expression in the hemizygous *rot1Δ*/*ROT1* strain was stable and comparable to the CAI4 control, regardless of doxycycline treatment.

### 3.3. Rot1 Influences N-Glycosylation in C. albicans

Previous studies in *S. cerevisiae* demonstrated that Rot1 physically interacts with the Ost3 subunit of the oligosaccharyltransferase (OST) complex, which mediates the transfer of oligosaccharides to nascent proteins [7]. However, it was unknown whether reduced *ROT1* expression in *C. albicans* could affect *OST3* at the transcriptional level.

**Figure 3:**
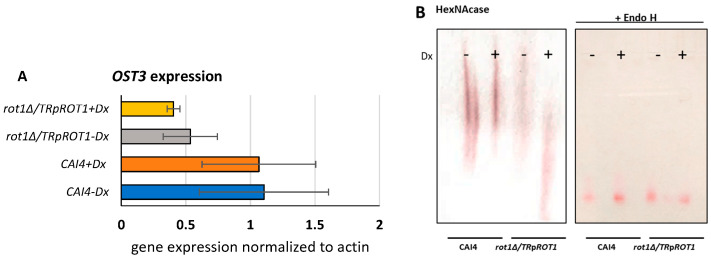
Rot1 influences *OST3* expression (**A**) and *N*-glycosylation of HexNAcase (**B**). The effect of Rot1 on *N*-glycosylation was analyzed in the CAI4 (control) and *rot1Δ*/*TR*p*ROT1* strains. Cells were cultivated under doxycycline-repressive (+Dx) or derepressive (−Dx) conditions to regulate *ROT1* expression. *N*-glycosylation of the model glycoprotein HexNAcase was assessed by changes in electrophoretic mobility under non-denaturing conditions. Where indicated, samples were treated with Endo H to remove *N*-linked glycans. Increased electrophoretic mobility of HexNAcase in the mutant strain under +Dx conditions indicates reduced *N*-glycosylation. Data represent mean ± SD from three independent experiments, each performed in triplicate.

RT-qPCR analysis revealed that *OST3* expression is strongly dependent on *ROT1* levels. In the *rot1Δ*/*TR*p*ROT1* strain, *OST3* transcript levels were reduced regardless of whether *ROT1* was overexpressed (−Dx) or repressed (+Dx), compared to the wild-type CAI4 strain (Figure 3A).

Given the essential role of Ost3 in *N*-glycosylation, we next examined the impact of altered *OST3* expression on the glycosylation of a model *N*-glycosylated protein, the hydrolytic enzyme *N*-acetylglucosaminidase (HexNAcase) [24,29].

Analysis of HexNAcase mobility by non-denaturing Tris/acetate 3–8% gradient PAGE revealed that doxycycline-induced repression of *ROT1* (+Dx) in the *rot1Δ*/*TR*p*ROT1* strain significantly reduced glycosylation, as indicated by increased electrophoretic mobility compared to CAI4 grown without doxycycline (−Dx) (Figure 3B). HexNAcase from the *rot1Δ*/*TR*p*ROT1* strain grown without doxycycline (overexpression) exhibited glycosylation levels similar to the control but appeared to be produced in lower amounts or displayed reduced enzymatic activity. These results indicate that limited Rot1 expression impairs *N*-glycosylation in *C. albicans*. After Endo H treatment to remove *N*-glycans, the HexNAcase migrates faster through the gel and appears as a single band.

**Figure 4:**
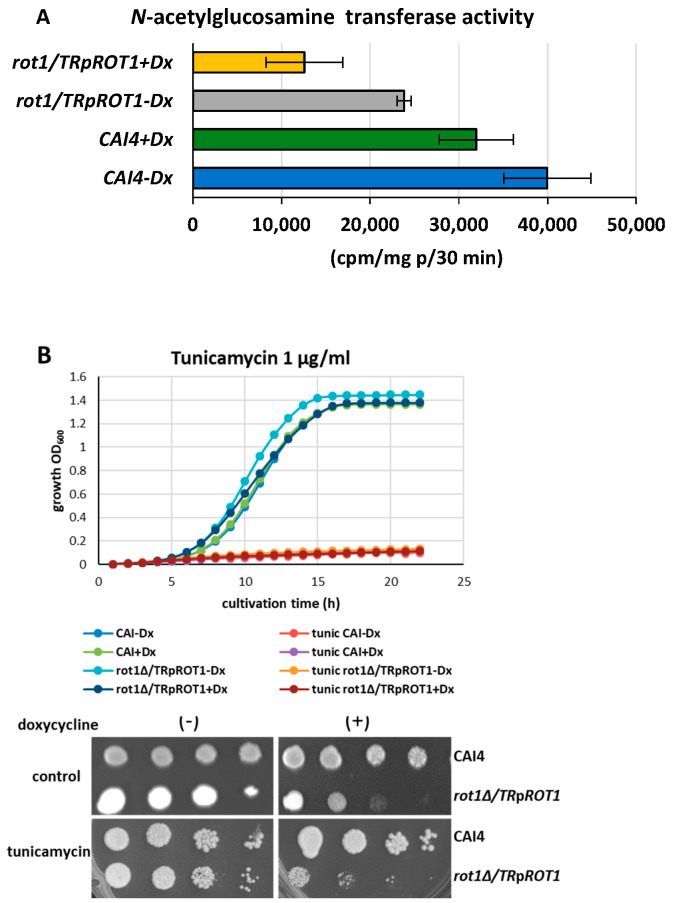
Activity of *N*-acetylglucosamine transferase (**A**) and tunicamycin sensitivity (**B**) of the *rot1Δ*/*TR*p*ROT1* mutant. Activity of *N*-acetylglucosamine transferase (Alg7/13) was analyzed in the *rot1Δ*/*TR*p*ROT1* mutant and the CAI4 control strain cultivated in the presence (+Dx) or absence (−Dx) of doxycycline. Sensitivity of the *rot1Δ*/*TR*p*ROT1* strain to tunicamycin was assessed by spot assay. Three microliters of a 10-fold serial dilution (starting from 1 × 10^7^ cells) of the indicated strains grown in liquid medium were spotted onto YPD agar plates supplemented with 40 µg/mL uridine (control) and 1 µg/mL tunicamycin. Data obtained from three independent experiments, each determined in triplicate.

Since oligosaccharide transfer to proteins by OST is impaired in the *rot1Δ*/*TR*p*ROT1* strain under doxycycline (+Dx), we hypothesized that upstream steps in lipid-linked oligosaccharide (LLO) synthesis might also be affected. To test this, we measured the activity of *N*-acetylglucosamine transferase (Alg7/13), the first enzymatic complex in the *N*-glycosylation pathway. Rot1 depletion corresponded to a 61% reduction in Alg7/13 activity compared to CAI4 (Figure 4A).

Given that Alg7 is inhibited by tunicamycin [30], we tested the sensitivity of *rot1Δ*/*TR*p*ROT1* to tunicamycin. The growth of the doxycycline-treated mutant was further reduced in the presence of 1 µg/mL tunicamycin (Figure 4B), and liquid culture experiments showed near-complete inhibition of both the mutant and control strains, regardless of doxycycline treatment. These results confirm that reduced Rot1 impairs early steps in *N*-glycosylation, enhancing susceptibility to inhibitors of LLO synthesis.

Finally, we assessed whether limited oligosaccharide transfer affects the expression of another enzyme, dolichyldiphosphate dephosphorylase Cwh8 [31].

**Figure 5:**
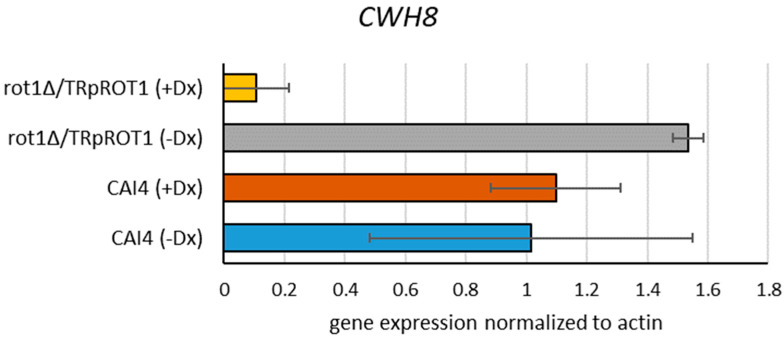
Expression of the *CWH8* gene encoding DolPP dephosphorylase in the *rot1Δ*/*TR*p*ROT1* and CAI4 strains cultivated with or without doxycycline. Data obtained from three independent experiments, each determined in triplicate.

RT-qPCR analysis revealed that *CWH8* expression was decreased by 90% in the *rot1Δ*/*TR*p*ROT1* strain grown with doxycycline compared to the CAI4 control (Figure 5). This suggests that Rot1 depletion not only impairs OST function but also alters downstream dolichol metabolism.

### 3.4. Rot1 Modulates Sensitivity to ER and Abiotic Stress

If Rot1 functions as an ER chaperone, its depletion is expected to impair cellular tolerance to conditions that induce ER stress, such as the accumulation of unfolded proteins. To test this hypothesis, the *rot1Δ*/*TR*p*ROT1* strain and the CAI4 control were exposed to dithiothreitol (DTT), which disrupts disulfide bonds in proteins and triggers ER stress.

**Figure 6:**
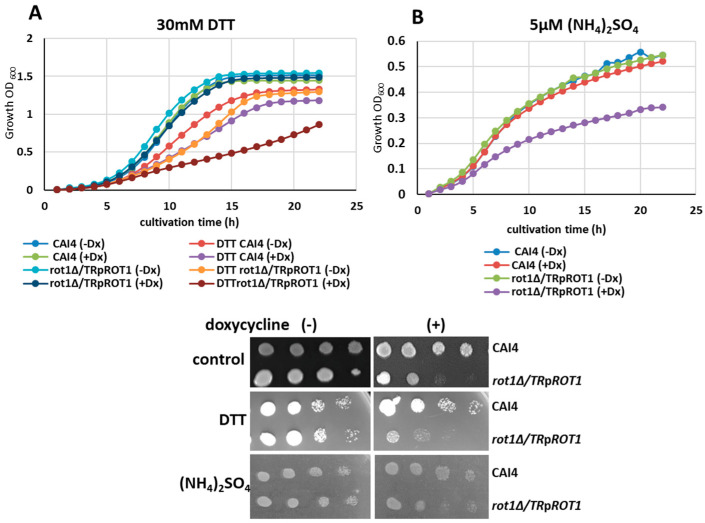
Sensitivity of *rot1Δ*/*TR*p*ROT1* and CAI4 strains to ER stress (**A**) and nitrogen limitation (**B**). Serial 1:10 dilutions (starting from 1 × 10^7^ cells) of indicated strains were spotted on: YPD + 40 µg/mL uridine (control); YPD + 30 mM DTT (ER stress); SD medium with 5 µM (NH_4_)_2_SO_4_ (nitrogen-limiting conditions). Data represent a representative experiment from three independent replicates.

Growth assays in the liquid YPD medium supplemented with 30 mM DTT revealed that, under derepressive conditions (−Dx), DTT inhibited growth of CAI4 and *rot1Δ*/*TR*p*ROT1* by 23% and 26%, respectively (Figure 6A). Under doxycycline-mediated *ROT1* repression (+Dx), the growth of the mutant strain was significantly more impaired than the control: inhibition reached 47% for *rot1Δ*/*TR*p*ROT1* compared to 29% for CAI4. These results demonstrate that Rot1 depletion increases susceptibility to ER stress, consistent with a role as an ER chaperone.

Given the reported cooperation of Rot1 with the TOR2 complex, which regulates growth in response to nutrient availability, we also examined sensitivity to nitrogen limitation. When the *rot1Δ*/*TR*p*ROT1* strain was cultivated with doxycycline in medium containing low ammonium sulfate (5 µM (NH_4_)_2_SO_4_), growth was reduced by 35% relative to CAI4 grown under the same conditions (Figure 6B). This finding suggests that Rot1 contributes to cellular adaptation to nutrient stress, in addition to its role in protein folding and ER homeostasis.

### 3.5. Rot1 Regulates Cell Wall Composition in C. albicans

Rot1 has been previously implicated in β-1,6-glucan synthesis and in activation of cell wall compensatory pathways, including increased chitin deposition [13]. To investigate the role of Rot1 in *C. albicans* cell wall integrity, we analyzed the carbohydrate composition of the *rot1Δ*/*TR*p*ROT1* mutant under derepressive (−Dx) and repressive (+Dx) conditions.

**Figure 7:**
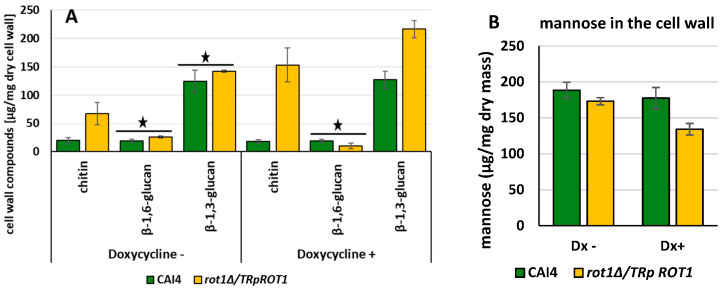
Carbohydrate composition (**A**) and mannose content (**B**) in the cell wall of CAI4 (wild type) and *rot1Δ*/*TR*p*ROT1* strains under doxycycline-repressive (+Dx) or derepressive (−Dx) conditions. Data represent mean ± SD from three independent experiments. Black stars indicate differences not statistically significant (*p* = 0.05).

Analysis revealed that the *rot1Δ*/*TR*p*ROT1* strain grown without doxycycline exhibited a 242% increase in chitin content relative to the CAI4 control. Surprisingly, repression of *ROT1* (+Dx) caused a further 742% increase in chitin, indicating that low Rot1 levels strongly trigger compensatory chitin synthesis (Figure 7A). Concurrently, β-1,6-glucan content decreased by 48% under *ROT1* repression, whereas overexpression (−Dx) led to a modest 34% increase compared to the control.

The β-1,3-glucan content also changed: under doxycycline repression, β-1,3-glucan increased by 70% in the *rot1Δ*/*TR*p*ROT1*, while overexpression (−Dx) resulted in a non-significant 14% increase relative to CAI4. Additionally, mannose content decreased by 23% in the doxycycline-treated mutant compared to the strain grown without doxycycline (Figure 7B). These results indicate that Rot1 depletion causes dramatic remodeling of the cell wall, with strong induction of chitin and β-glucan compensatory mechanisms and a reduction in mannose content.

To determine whether these compositional changes affected cell wall stability, we tested the sensitivity of the mutant to cell wall-perturbing agents Calcofluor White (CFW) and Congo Red (CR).

**Figure 8:**
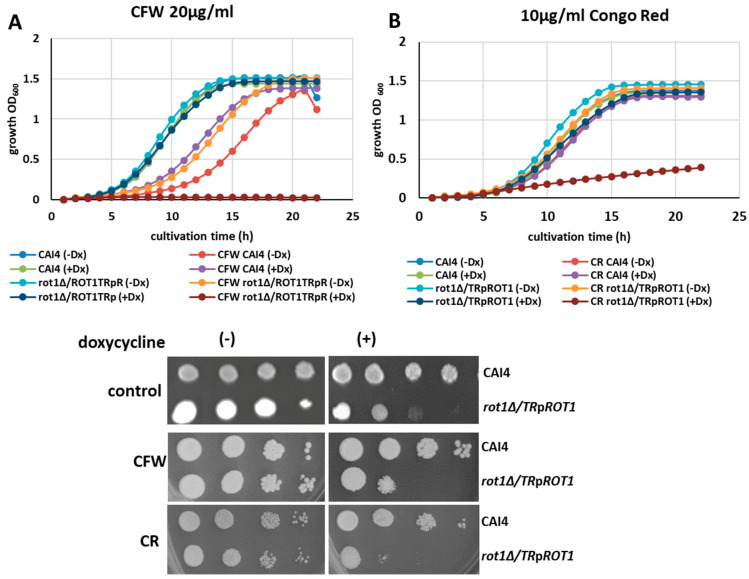
Sensitivity of CAI4 and *rot1Δ*/*TR*p*ROT1* strains to Calcofluor White (CW, 20 µg/mL) (**A**) and Congo Red (CR, 10 µg/mL) (**B**). Serial 1:10 dilutions (starting from 1 × 10^7^ cells) were spotted on YPD plates supplemented with 40 µg/mL uridine (control), CFW, or CR and incubated at 30 °C for 72 h. Doxycycline (+Dx) significantly increased the sensitivity of the mutant to both agents.

Growth assays showed that CFW at 20 µg/mL completely blocked growth of the *rot1Δ*/*TR*p*ROT1* strain under doxycycline-induced *ROT1* repression, while CR (10 µg/mL) also caused significant growth inhibition (Figure 8A,B). These findings confirm that Rot1 is essential for maintaining cell wall integrity in *C. albicans* and that its depletion renders cells highly susceptible to cell wall stress.

### 3.6. Altered Cell Wall Composition Increases Sensitivity to Caspofungin

Caspofungin inhibits β-1,3-glucan synthesis in the *Candida* cell wall and induces compensatory chitin accumulation [32]. Since the *rot1Δ*/*TR*p*ROT1* strain grown with doxycycline exhibits very high chitin levels and elevated β-1,3-glucan, we assessed whether this strain remains sensitive to caspofungin.

**Figure 9:**
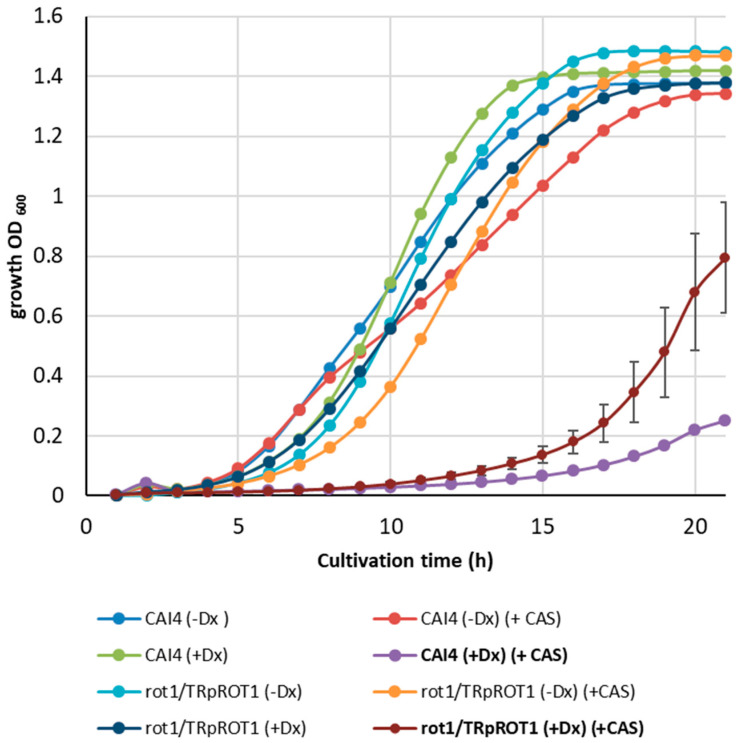
Sensitivity of CAI4 and *rot1Δ*/*TR*p*ROT1* strains to caspofungin (CAS, 1 mg/L) under doxycycline-repressive (+Dx) or derepressive (−Dx) conditions. Growth inhibition (%) was calculated relative to untreated controls after 15 h.

Growth assays in YPD medium supplemented with 1 mg/L caspofungin showed that the CAI4 control strain was almost completely inhibited under doxycycline conditions, with growth reaching only 5.8% of untreated control, whereas in the absence of doxycycline, inhibition was modest (26% after 15 h of cultivation) (Figure 9).

The *rot1Δ*/*TR*p*ROT1* strain displayed a similar pattern: under *ROT1* repression (+Dx), caspofungin reduced growth by 86%, while without doxycycline (−Dx) only 11% growth inhibition was observed. These results indicate that Rot1 depletion alters cell wall composition, and the combined stress of doxycycline-induced *ROT1* repression and caspofungin treatment significantly impairs cell growth. Interestingly, the effect of doxycycline plus caspofungin was more pronounced in CAI4 than in the mutant, suggesting that the mutant’s altered cell wall partially mitigates drug action, likely due to already elevated chitin levels.

### 3.7. Rot1 Is Required for Filamentation in C. albicans

**Figure 10:**
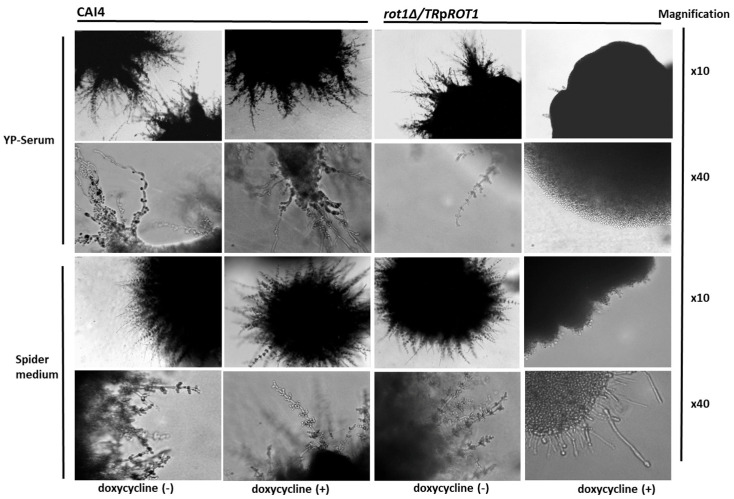
Hyphal growth of CAI4 and *rot1Δ*/*TR*p*ROT1* strains on YP-Serum and Spider medium with or without doxycycline. Colonies were photographed after 7 days at 30 °C using light microscopy. Magnification is indicated.

To determine the impact of *ROT1* expression on morphogenesis, hyphal formation was induced for 7 days on YP + 10% serum medium and Spider medium at 30 °C, with or without doxycycline. Colonies were examined under a light microscope (Figure 10).

The *rot1Δ*/*TR*p*ROT1* mutant failed to form filaments under doxycycline repression, although residual hyphae were visible when grown on Spider medium. The same strain under derepressed conditions (−Dx) and the control strain CAI4 showed vigorous hyphal growth on both media. This indicates that Rot1 is required for filamentation, a key virulence trait in *C. albicans*.

## 4. Discussion

The primary goal of this study was to clone and functionally characterize the Rot1 protein from *C. albicans*. Previous knowledge of Rot1 was largely limited to *S. cerevisiae*, where it has been shown to influence *N*- and O-glycosylation, dolichol synthesis, cell wall assembly, and protein folding, and deletion of *ROT1* is lethal.

A homolog of ScRot1 was identified in *C. albicans* (orf19.6029, hereafter Ca*ROT1*), and its function was confirmed by complementation in an *S. cerevisiae Δrot1*/*ROT1* mutant. In *C. albicans*, a conditional *rot1Δ*/*TR*p*ROT1* strain allowed us to study the consequences of reduced *ROT1* expression.

A major focus was to assess the potential role of Rot1 in pathogenic traits, particularly hyphal formation. Morphological switching from yeast to filamentous forms is central to *C. albicans* pathogenicity and depends on cell wall remodeling [33,34,35]. Our data show that reduced *ROT1* expression caused major alterations in cell wall composition, including decreased β-1,6-glucan and mannose, compensated by elevated β-1,3-glucan and chitin. Interestingly, these changes resembled those observed in hyphal cell walls, even though the cells were in yeast form [36,37].

Cell wall composition can significantly influence antifungal drug activity. Caspofungin alone had no effect on the growth of CAI4 and rot1Δ/TRpROT1 strains. However, when doxycycline was added to caspofungin-containing medium, growth inhibition was observed in both strains. A synergistic interaction between these two agents was previously described by Miceli et al. [38]. Notably, the CAI4 strain exhibited greater sensitivity to this synergistic effect than the rot1Δ/TRpROT1 strain.

Caspofungin acts as a noncompetitive inhibitor of β-1,3-glucan synthase, thereby disrupting fungal cell wall synthesis [39]. The rot1Δ/TRpROT1 strain, however, contains elevated levels of chitin in its cell wall. Increased chitin is known to compensate for β-1,3-glucan synthase inhibition and can confer reduced susceptibility to echinocandins such as caspofungin [40].

Despite these compensatory alterations in cell wall composition, the rot1Δ/TRpROT1 strain was unable to form hyphae. This defect suggests that proper glycosylation—regulated in part by Rot1—is essential for filamentation. Therefore, although enhanced chitin content may partially protect against cell wall–targeting drugs, correct glycosylation remains critical for maintaining morphogenetic processes such as hyphal development [14,15,16,17].

Indeed, limited *ROT1* expression inhibited *N-*glycosylation, as evidenced by underglycosylation of the marker protein HexNAcase. This deficiency caused a feedback inhibition of lipid-linked oligosaccharide (LLO) synthesis, reducing the activity of *N*-acetylglucosamine transferase (Alg7/13) and downregulating DolPP dephosphorylase (Cwh8). These effects prevent accumulation of unutilized oligosaccharides in the ER, indicating a tight regulatory network linking Rot1, glycosylation, and ER homeostasis.

Consistent with its proposed chaperone function, Rot1 was critical for survival under ER stress induced by DTT. Moreover, the *rot1Δ*/*TR*p*ROT1* strain exhibited reduced growth under nitrogen-limiting conditions, consistent with Rot1’s known connection to TOR2 signaling in *S. cerevisiae* [2,41].

Taken together, these results demonstrate that Rot1 in *C. albicans* is a multifunctional protein: it regulates glycosylation, cell wall integrity, ER stress tolerance, and nutrient response. Proper function of these processes is essential for yeast-to-hyphae transition. The pleiotropic nature of Rot1 suggests that its absence disrupts multiple interconnected pathways, preventing filamentation and, by extension, the formation of invasive structures.

Thus, Rot1 can be considered a “superprotein”, integrating multiple cellular processes that collectively support pathogenicity in *C. albicans*. Since Rot1 has no human homolog, it represents an attractive potential target for antifungal therapy.

## 5. Conclusions

CaRot1 is an essential regulator of protein *N*-glycosylation, LLO synthesis, and ER function in *C. albicans.*

Rot1 maintains cell wall integrity, controlling β-glucan, chitin, and mannose levels, and modulates sensitivity to antifungals and cell wall stressors.

Rot1 is critical for filamentation, linking proper glycosylation and cell wall remodeling to pathogenic morphogenesis.

Overall, Rot1 functions as a “superprotein”, coordinating multiple processes that collectively support *C. albicans* growth, stress tolerance, and virulence.

## Data Availability

The original contributions presented in this study are included in the article/Supplementary Material. Further inquiries can be directed to the corresponding author.

## Supplementary Materials

The following supporting information can be downloaded at https://www.mdpi.com/article/10.3390/jof12040244/s1, Figure S1: Southern blot analysis of *ROT1* mutated strains; Table S1: Primers used for strain construction.
