# Supplementary material for: The Essential Rot1 Protein Links Glycosylation, Cell Wall Integrity, and Pathogenic Development in Candida albicans
Source: J Fungi (Basel). 2026 Mar 26;12(4):244. doi: 10.3390/jof12040244 (PMC13117340; doi:10.3390/jof12040244)
Supplement: Supplementary file 1 [file jof-12-00244-s001.zip › jof-4179337-supplementary.pdf]

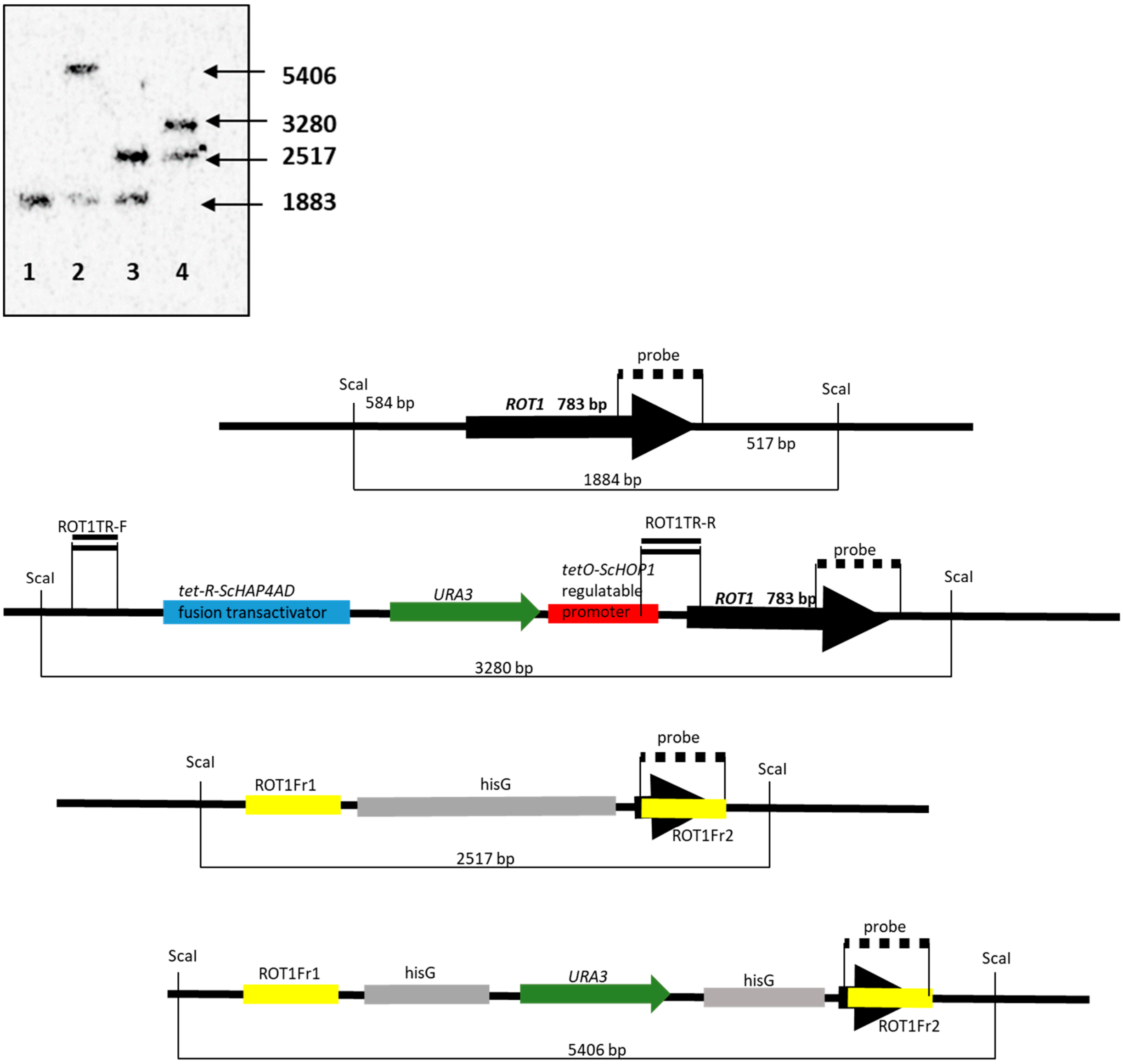

Figure S1. Southern blot analysis of *ROT1* mutated strains.
1. wild type CAI4 strain; 2. hemizygote *rot1:: hisG-URA3-hisG/ROT1*; 3. *rot1::hisG/ROT1* (*rot1Δ/ROT1*); 4. *rot1::hisG/TR*p*ROT1* (*rot1Δ/TR*p*ROT1*)
DNA from all the strains was cut with ScaI and hybridized with a 333-bp probe homologous to the orf.196029 coding region. The analysis revealed one band represented two wild copies of the *ROT1* (1883 bp) in the CAI4 parental strain (line1). Two bands were hybridized in the hemizygote *rot1:: hisG-URA3-hisG/ROT1* lower band of 1883 bp representing untouched *ROT1* copy and the upper band of 5406 bp is a disrupted *ROT1* copy of the gene containing *hisG-URA3-hisG* sequence. Line 3 representing wild copy of the *ROT1* (1883 bp) and disrupted *rot1::hisG* copy of the gene (2517 bp). Line 4 – the upper band (3280 bp) representing *ROT1* gene under the TR promoter.

Table S1.

Primers used for strain construction

Primer name	5′-sequence-3′	Purpose	Remarks
ROT1orfF	GATCCATGATATTTTCGAGGTATTTTATTC	Synthesis of CaROT1 from the ATG to the STOP codons	BamHI site underlined
ROT1orfR	GCTAGCTTATCTTTTAAGAAAGAAATATGATGA		NheI site underlined
mutROT1F	TCAGAAACTGCCTCGACTTTAGGTA	Mutation of the CTG codon to TCG in *CaROT1*	
mutROT1R	TACCTAAAGTCGAGGCAGTTTCTGA		
ROT1Fr1F	TGAGCTCTTGATTTCTTCTGTTTATTGC	Synthesis of flank 1 for construction of cassette for URA-Blaster deletion	SacI site underlined
ROT1Fr1R	TACAGATCTCGACGAATAATGGTGATG		BglII site underlined
ROT1Fr2F	TATGTCGACTTTAGGTAGTGTGTCTCA	Synthesis of flank 2 for construction of cassette for URA-Blaster deletion	SalI site underlined
ROT1Fr2R	TATCTGCAGCAAGAACATTCAACCCTA		PstI site underlined
RotVerF	GTAAATTGGTAGGGCGTGCA	Detection of proper *URA3* cassette integration	
VerhGR	GCGCTTTCAGTTTCTCCATG		
ROTtetF	GAAACTTCACGAGTTCCTTATACATACTTGTCATTTTCTTCCTACTAAAGCTTACCAAAAAAAGATATAGGGCGAATTGGGTAC	Construction of cassette for *C. albicans* transformation with TRp promoter	Fragment homologous to TRp amplified on p2151c plasmid underlined
ROTtetR	ACGAATAATGGTGATGATGTTAAAAGTGT GAGTATAATAGGAATAAAATACCTCGAAAATATCATCTAGTTTTTCTGAGATAAAGCTG		
TetVerF	GTCAAGTCTCCAATCAAGGT	Verification of proper integration of TRp cassette	
TetRotR	CTAAAGTCAGGGCAGTTTCT		
